# The importance of dietary change for men diagnosed with and at risk of prostate cancer: a multi-centre interview study with men, their partners and health professionals

**DOI:** 10.1186/1471-2296-15-81

**Published:** 2014-05-03

**Authors:** Kerry NL Avery, Jenny L Donovan, Jeremy Horwood, David E Neal, Freddie C Hamdy, Chris Parker, Julia Wade, Athene Lane

**Affiliations:** 1 School of Social and Community Medicine, University of Bristol, 39 Whatley Road, Clifton, Bristol BS8 2PS, UK; 2 Oncology Centre, Addenbrooke’s Hospital, Hills Road, Cambridge CB2 0QQ, UK; 3 Nuffield Department of Surgical Sciences, University of Oxford, John Radcliffe Hospital, Headley Way, Oxford OX3 9DU, UK; 4 Academic Urology Unit, Royal Marsden NHS Trust, Institute of Cancer Research, Sutton, Surrey, SM2 5PT, UK

**Keywords:** Cancer, Diet, Oncology, Prostatic neoplasms, Qualitative research, Survivors

## Abstract

**Background:**

The diagnosis of prostate cancer (PC) can provide a trigger for dietary change, and there is evidence that healthier diets may improve quality of life and clinical outcomes. However, men’s views about dietary change in PC survivorship are largely unknown. This multi-centre qualitative interview study explored men’s views about dietary change in PC survivorship, to better understand motivations for, and barriers to, achieving desired changes. The role of radical and active surveillance treatments on dietary change and the influence of men’s partners were examined. Focus groups also evaluated stakeholder opinion, including healthcare professionals, about the provision of dietary advice to PC patients.

**Methods:**

A multi-centre interview study explored views about diet and motivations for, and barriers to, dietary change in men at elevated risk or diagnosed with PC following prostate specific antigen (PSA) testing. 58 men and 11 partners were interviewed. Interviews and focus groups were undertaken with 11 healthcare professionals, 5 patients and 4 partners to evaluate stakeholders’ opinions about the feasibility and acceptability of providing dietary advice to PC patients. Data were analysed using methods of constant comparison and thematic analysis.

**Results:**

Over half of diagnosed men reported making dietary changes, primarily to promote general or prostate health or facilitate coping, despite their uncertainty about diet-PC links. Interest in dietary advice was high. Information needs varied depending on treatment received, with men on active surveillance more frequently modifying their diet and regarding this as an adjunct therapy. Men considered their partners integral to implementing changes. Provision of dietary advice to men diagnosed with PC was considered by healthcare professionals and men to be feasible and appropriate in the context of a holistic ‘care package’.

**Conclusions:**

Many men make positive dietary changes after PC diagnosis, which are perceived by men and their partners to bring psychological and general health benefits and could help future dietary intervention trials. Men and their partners desire more and better dietary information that may support PC survivorship, particularly among those embarking on active surveillance/monitoring programmes. There are opportunities for healthcare professionals to support PC patients both clinically and psychologically by the routine integration of healthy eating advice into survivorship care plans.

## Background

Prostate cancer (PC) is the second most common cancer in men and incidence rates may double by 2030 [1]. With increased awareness and levels of prostate specific antigen (PSA) testing and improved treatment strategies, the number of men living with the disease is also rising [2]. Growing evidence indicates that a healthy diet may improve overall clinical outcomes [3,4] and quality of life [5,6] in cancer, with the strongest evidence regarding PC for a protective effect from foods containing lycopene, a component of tomatoes [3].

Some previous research indicates that around half of men change their diet after PC diagnosis [7-9]. However, the factors motivating or preventing men at elevated risk or diagnosed with PC from achieving desired changes, and their attitudes about dietary advice, are poorly understood. It is recognised, furthermore, that social support can assist healthy eating and successful dietary change in patients with other diseases (e.g. cardiovascular disease, Type II diabetes) [10,11]. There has been no empirical interview research comparing the impact of different radical and non-radical PC treatments on men’s dietary decisions. Identifying facilitators and obstacles to dietary change may also inform the design of dietary intervention trials, where initiating and sustaining dietary changes is frequently problematic and difficulties with compliance and attrition often arise [12]. The views of stakeholders (e.g. patients and their partners, healthcare providers and researchers) about the provision of dietary advice to men diagnosed with PC are uncertain and men’s experiences and perceptions of being given dietary information after a PC diagnosis are also uncertain. This interview study explored views about diet and motivations for and barriers to dietary change in men at elevated risk and those diagnosed with PC. This included the possible role of different radical and non-radical treatments received and the influence of men’s partners on dietary change. Subsequent interviews and focus groups with healthcare professionals, patients and their partners also sought to evaluate stakeholders’ opinions about the feasibility and acceptability of the routine provision of dietary advice to PC patients within the NHS.

## Methods

### Sampling

The aim was to include a wide range of participants, including patients, partners and health professionals from different settings and with different experiences of prostate cancer diagnosis, survivorship or healthcare. Several sampling strategies, including maximum variation purposive (non-probability) sampling, were used to ensure the inclusion of the range of characteristics of the population, including the recruitment of men participating in the ongoing ProtecT (Prostate Testing for Cancer and Treatment) randomised treatment trial [13]. Data derived from food frequency questionnaires (FFQ) completed by ProtecT men before and one year post-diagnosis were also used to ensure participants were sampled across the range of degrees of dietary change.

### Participants and recruitment

**Figure 1:**
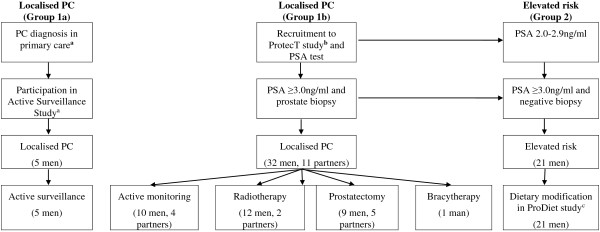
**Flow diagram of study recruitment. **^a^Royal Marsden Hospital, ^b^University of Bristol, ^c^University of Bristol, dietary modification comprised a lycopene component (active capsules, placebo capsules or 1–2 portions of cooked tomatoes per day) and a green tea component (active capsules, placebo capsules or 3 cups of green tea per day).

There were three sets of participants (Figure 1 and Table 1):

(i) **Men diagnosed with PC and their partners in two settings:** The first group were recruited from the ProtecT trial. In ProtecT, men registered with primary care centres in nine UK areas aged 50–69 with no history of PC received mailed information and a recruitment clinic appointment for a PSA test. Men with a raised PSA result (≥3.0 ng/ml) were invited for transrectal ultrasound-guided biopsy and those diagnosed with clinically localised disease were offered randomisation to radical prostatectomy, conformal external beam radiotherapy or active monitoring (AM), a form of surveillance that comprised regular scheduled assessments of PSA levels and disease status and is comparable to active surveillance (AS - close monitoring of prostate specific antigen levels combined with periodic imaging and repeat biopsies) without scheduled biopsies. For the purposes of this qualitative study, ProtecT men and their partners were interviewed at varying timepoints after diagnosis. The second group were men diagnosed with localised PC by their primary care centre and who subsequently participated in a study investigating clinical outcomes of AS (the ‘Active Surveillance Study’, Royal Marsden Hospital, UK). A sample of these men was interviewed after commencing AS.

(ii) **Men at elevated risk of PC:** Men were recruited from the nested ProDiet study, which was investigating the feasibility of recruiting men from the ProtecT study with a PSA level of 2.0-2.9 ng/ml or ≥3.0 ng/ml with a negative biopsy into a randomised dietary modification trial. Men who consented were randomised to both a lycopene (active capsules, placebo capsules or 1–2 portions of cooked tomatoes per day) and a green tea dietary component (active capsules, placebo capsules or 3 cups of green tea per day) for six months. A sample of these men was interviewed immediately after randomisation.

(iii) **Stakeholders:** A stakeholder evaluation, using focus groups and interviews, was undertaken to explore views about the routine provision of dietary advice to men diagnosed with or at elevated risk of PC. Key stakeholders were invited, including 18 service providers (healthcare professionals and support group workers) identified from local hospitals, academic institutions and primary care practices, 9 men diagnosed with PC in the ProtecT study and 5 of their female partners.

**Table 1:** Participant characteristics (n = 69)

	**Localised PC**^**a **^**(n = 37)**	**Elevated risk**^**b **^**(n = 21)**	**Partners**^**c **^**(n = 11)**
**Mean age, years (range)**	66.5 (54.4– 75.4)	65.4 (52.5 – 72.3)	65.1 (59.8 – 72.2)
**Mean PSA level, ng/ml (range)**	5.6 (3.0 – 12.9)	3.0 (2 – 9.2)	N/A
**Treatment, n**	-	N/A	-
Active monitoring/surveillance	15		4^d^
Radical radiotherapy	12		2^d^
Radical prostatectomy	9		5^d^
Brachytherapy	1		0^d^
**Marital status, n**			
Married/living as married	31	21	11
Not married	6	0	0
**Occupational class**^**e**^**, n**			
Managerial & professional	13	11	2^d^
Intermediate	8	3	3^d^
Working	11	7	6^d^

N/A = not applicable, PSA = Prostate Specific Antigen, ^a^including 32 men in ProtecT study and 5 men in AS study, ^b^PSA 2.0-2.9 ng/ml or ≥3.0 ng/ml with a negative biopsy, included in ProDiet study, ^c^of ProtecT participant, ^d^of ProtecT man, ^e^based on participant’s occupation using the three-class system [35], not available for Active Surveillance Study participants.

### Data collection

Data collection comprised two phases.

**Phase 1 - Patient and partner interviews:** The aim of these interviews was to explore participants’ views about prostate cancer and diet. Of the 61 men and 11 partners of men invited for interview by the study researcher (KA) between January 2008 and March 2012, 69 (58 men and 11 female partners; 96%) agreed (Figure 1). Interviews were conducted between 7–42 months post-diagnosis and lasted 9–90 minutes. Partners were interviewed simultaneously or separately depending on the couples’ preferences. Interviews were semi-structured using topic guides informed by literature review and discussion between study researchers. Interviews were conducted in the participant’s home (n = 46) or by telephone (n = 23).

**Phase 2 - Stakeholder evaluation:** Interviews and a focus group were undertaken with key stakeholders to examine their interpretation of and opinions towards current evidence linking prostate cancer to diet, and their attitudes and preferences about the provision and communication of dietary advice post-diagnosis. Of the 9 men/partners invited, a focus group was conducted with 5 men diagnosed with PC in the ProtecT study (response rate 56%, mean age 68.3 years) and 4 of their partners (response rate 80%, mean age 68.0 years). Of the 18 invited, in-depth interviews were conducted with 11 service providers (61%, 6 male, 5 female, mean age 46.2 years), including Consultant Urologists/Oncologists (n = 4), dieticians (n = 3), public health nutritionists, primary care practitioners, Uro-oncology specialist nurses and prostate cancer support group worker (one of each). Participants were presented with a summary of the World Cancer Research Fund’s (WCRF’s) Expert Report on food, nutrition and PC prevention [3], the most recent summary of the literature to date, during the focus group. A topic guide was used to evaluate the extent of stakeholders’ existing knowledge about the role of diet in PC prevention prior to being presented with a summary of the WCRF report and attitudes about the provision of dietary advice to PC patients. The focus group lasted 107 minutes and the interviews 19–52 minutes.

### Analyses

Interviews and the focus group were conducted by KA, audio-recorded, transcribed and analysed using NVivo9 software. Analyses were conducted by KA to inductively identify recurring themes (concepts or explanatory ideas) from the data, via line-by-line coding of textual data, and to examine relationships between them [14,15]. Themes were then assigned brief descriptive codes. After transcripts had been coded, emerging themes were compared with newly-collected data to test and refine themes [16]. Where appropriate, themes were categorised to facilitate higher-level interpretation of the data. Sampling and analyses continued until no new themes were emerging and established themes ceased evolving. A subset of interviews was independently analysed by a second researcher (JH, JW) and coding discrepancies discussed to maximise rigour and reliability. Plausibility of data interpretation was further discussed between the study team throughout the analyses (JD, AL, JW, KA) and ‘negative’ cases, in which respondents’ perspectives or experiences differed from the main body of evidence, were sought to explore participants’ divergent views and to enhance the credibility of the analyses and results [16]. Quotations from a range of respondents are provided below to illustrate themes that emerged during the analyses where the prefix P*n* denotes a ProtecT participant, F*n* his partner, D*n* a ProDiet participant, A*n* an Active Surveillance study participant and S*n* a service provider or healthcare professional participant. The characters … denote omitted text. Ellipses ( … ) contain a reference to a person’s name.

Ethical approval for this interview/focus group study, the ProtecT study and the ProDiet study was obtained from Trent Multi-centre REC (MREC/01/4/025–21/06/2001, 09/H0405/37–12/11/2009 and ProDiet 08/H0405/61) and for the AS study from the Royal Marsden REC. Written or verbal informed consent was obtained from all participants.

## Results

Interviews with men at elevated risk or diagnosed with PC and their partners were conducted with participants from a range of age groups, trial recruiting centres, pre-diagnosis PSA levels and treatments (Table 1). Reflecting the demographics of the studies from which participants were sampled, most (68, 98.6%) were from White ethnic groups.

### Men’s perceptions of the cause of prostate cancer

Men at elevated risk or diagnosed with PC typically expressed uncertainty about causes of PC and the potential role of diet in PC aetiology. Consequently, men rarely attributed their disease to dietary causes: “I think there are many other factors which are much more important … for example genetic background, I think all have a much more significant role than things like diet” (P3).

### Triggers, facilitators and barriers to dietary change

Men were mostly uncertain about whether diet could help manage or prevent progression or recurrence of disease. However, over half (22) of the 37 men with PC reported changing their diet post-diagnosis, most commonly eating more tomatoes/tomato products, more fruits and vegetables or decreasing fat intake. Fifteen men had improved their diet previously, e.g. after experiencing high cholesterol, but a PC diagnosis often served as a ‘wake-up call’ or extra incentive to consider dietary improvements to enhance their general and, subsequently, their prostate health (see the ‘List of examples of reasons for dietary change in men diagnosed with PC’). Dietary change also served as a coping strategy for some men, with four describing feeling they were ‘doing something’ to help and two explaining that focusing on dietary changes helped them manage their uncertainty and regain some control over their disease and their future survivorship. Similar feelings were expressed by men at elevated risk.

List of examples of reasons for dietary change in men diagnosed with PC

PC diagnosis as an incentive

P4: I can’t say it was definitely because of the prostate I think we had already decided before that was even diagnosed that we would try and eat a little bit healthier shall we say you know I’m 60 now …

Maintaining general health after diagnosis

P30: … I think it’s just the general thing just to look after your health through eating healthily. How much it’s related to any cancer I’ve no idea really.

Preventing progression and/or recurrence or to be ‘fighting fit’ for further treatment

P7: Well to restrict the cancer from spreading as much as anything.

F6: … no I don't think it's (diet) going to prevent cancer no … I just think it probably reduces the risks … to generally, sort of, keep yourself healthy so you've got the best chance if it recurs.

Curing PC

P14: (the author of a published anti-cancer diet) had her cancer gradually diminished and I was hoping that would be the same with mine … curing it …

Preventing other cancers

P11: … you just hope that it will, well one make you more healthy … and two, I suppose hopefully reduce risks, um, whatever they are in the future for any other cancer I suppose.

Positive psychological effects of ‘doing something’

P11: … you feel more positive I think, you know, you sort of, you're doing something, you know.

P5: … I suppose it could be classified partly psychological, it’s doing some good … the hope it was all going to help.

P22: … my established method of managing uncertainty is to find out about it … that’s where I get my control back, I manage my emotions by being more rational it’s a denial strategy … an avoidance strategy.

Diet believed to cause PC

P24: … physically I earned it (the PC diagnosis) … I ate all the wrong things and drank all the wrong things .... And that’s when I embarked upon the programme that being very choosy about what I eat ....

Receiving dietary advice/information

P1: … I did read that, tomatoes, fresh tomato juice is good for so I was on that for quite a while.

P20: Well you hear snippets of information about what you should be eating and drinking … anyway I stopped taking copious amounts of orange juice and changed to cranberry and pomegranate juice.

Receiving information (e.g. from a family member, health professional, media) about potential diet-PC links triggered six PC patients to make changes. Irrespective of whether they made changes, most men expressed confusion and dissatisfaction with available dietary information and/or its contradictory nature. Notably, most non-changers of diet (11 out of 15 men) described unreliable information as a barrier to making dietary changes.

### Perceived relationship between PC treatment and dietary change

In men diagnosed with PC, treatment type, particularly its perceived success, greatly influenced attitudes toward potential benefits of dietary changes post-diagnosis (see the ‘List of examples of the impact of PC treatment on dietary change’). Men who received radical treatment (radiotherapy, prostatectomy or brachytherapy) were more likely to describe their treatment as ‘successful’, and their PC as ‘cured’ (P4,8,10,11,13), than those on AM or AS. Consequently, these men more often regarded dietary changes as unlikely to be beneficial, with a number disclosing that they may have considered changing their diet more had they not had radical treatment. In contrast, men on AM or AS were slightly more likely to change their diet (10/15 men on AM versus 5/9 men who had surgery and 6/12 of men who had radiotherapy). Men on AM or AS more often regarded dietary changes as an adjunct therapy. Some described positive psychological effects associated with ‘doing something’ potentially beneficial to manage/control their disease. This view was also shared by some men who underwent radiotherapy, with two reflecting that they may have changed their diet less had they received ‘curative’ surgery.

List of examples of the impact of PC treatment on dietary change

Dietary change unnecessary due to ‘curative’ treatment

P11 (surgery): … I've just parked it. I’ve had it done. I’ve dealt with it …

P4 (surgery): … if I had taken part in doing (radiotherapy or active monitoring) I think I would have been a bit more conscious of what I would eat and what I wouldn’t eat to try and keep it at bay … the fact that I’ve had the prostate out, that’s at the back of my mind really … so I’m living a normal life …

P9 (radiotherapy): Well I’m not really expecting it to come back again anyway so therefore do I have to change my diet …

P1 (radiotherapy): … now that I’ve got rid of my cancer … I suppose there’s a feeling that I don’t have to control my cancer … (had I been on active monitoring) I would have tried everything then of course … I would probably be drinking a gallon of tomato juice a day cos they say it’s good for you.

P12 (active monitoring): I don’t know if there would be any need to take the lycopene … if the prostate had been removed.

Dietary change as an adjunct therapy to active monitoring and radiotherapy

P12 (active monitoring): … it’s something positive isn’t it, you know, taking something, so it might help. … at the moment I’m on active monitoring, so as I isn’t being checked … I’m getting no other treatment am I? … I just felt that by sort of, if I can do anything that might help slow it up then I would.

P7 (active monitoring): So I’ve changed my diet, seen how that’s gone and initially my PSA reading dropped down … and then come back up … and so I seem to sort of hit a limit on what I can do with just tomatoes and now I’m going to be looking at alternative supplementation to see if there is something in there that would help (reduce) the cancers.

P30 (radiotherapy): … I was getting medical treatment so diet seemed to be the only thing I could do. I thought it’s no good having treatment and eating all types of food so I sort of had the idea that one negates the other …

Nothing to lose

P24 (radiotherapy): Whether (dietary change is beneficial) at such a late stage as I am, already having been diagnosed and treated I’m not sure about that but I work on the principal what harm can it do?

### Influence of men’s partners on dietary change

Men’s partners played a significant and multi-faceted role in their diets (see the ‘List of examples of the influence of partners on men's diet and men's dietary change’) and men often used plural terminology (e.g. ‘we’, ‘us’) referring to dietary decisions being made jointly. Whilst partners more often assumed responsibility for food purchasing and preparation, decisions about meal choices were mostly jointly made. Consequently, men typically described having considerable control or responsibility over their diet. Decisions to change diet were also often made jointly, though men’s partners were most commonly responsible for implementing these by buying food and preparing meals. Only three men (P4, P9, P11) acknowledged that dietary change was initiated solely by their partner but apparently not against their wishes. One man (P9), for example, reported that he would not have started taking selenium tablets had his wife not initiated this. The nature of dietary changes made was also sometimes influenced by their partner’s food preferences (e.g. a partner’s dislike of red meat helped one man to successfully reduce his intake). Regardless of who initiated dietary changes, the role men’s partners played in implementing and maintaining dietary changes often reduced men’s perception of responsibility in the process, making change easier. Some women explained how they would consider influencing their partner’s diets should dietary advice for PC become available. Some men (e.g. P11) felt that women are more knowledgeable about diet in relation to health, partly because dietary information is targeted at and more readily available to women in the media (e.g. in magazines). Only one woman (F1) expressed concern that their husband would be unwilling to change his diet.

List of examples of the influence of partners on men's diet and men's dietary change

The use of plural terminology

P7: We’ve tried, yep we do try to eat more fruit, again partly with the eye on the prostate cancer thing, yes.

Joint decision-making regarding dietary decisions and dietary change

P12: … they (the ProtecT study nurses) told like they told me at certain meetings (PSA check clinics) that some things are good and then I come home and tell (my wife) and then we try and implement it…

P1: I think it (changing diet) would be quite an easy thing to do, um, although I would have to programme my wife to stop buying the things that I like.

Partner-driven dietary changes

P11: Oh ((patient’s wife)) would, um, keep nudging me like: oh you should eat, you know, less of that or. No, I think certainly, uh, I think the, um, the woman in the house I think is a lot more switched on to things like that.

P4: … I mean the wife not really pressurised, she just sort of said oh you know maybe we ought to do a little bit differently.

P11: … I don’t make the effort but I don’t have to because… it’s being done for me. And so it makes it easier.

Mechanisms of bringing about dietary change

F4: I think it’s (dietary decisions) a joint thing but I think if I’d read somewhere that he definitely had to eat something, I would make sure that he ate it by hook or by crook like I did with the boys, I’d mix it in with the potatoes or something.

### Interest in dietary advice and information following diagnosis

Interest in dietary advice was high among men both at elevated risk and men diagnosed with PC, and their partners. Information needs post-diagnosis varied somewhat according to men’s perceptions of their treatment success and disease status, with one man on AM explaining “well I have prostate cancer, I would probably pay strong attention to (dietary advice)” (P7) and another who felt cured after radiotherapy requesting dietary information that “would make a difference to my well being … now that I am hopefully cancer free, I want to stay that way …” (P1). Only one man considered dietary advice irrelevant because he considered himself ‘cured’.

Men said they would welcome scientific and evidence-based dietary advice that became available: “… if it came from a reliable source and it weren’t just a maybe will you try this just in case” (P26). For dietary advice to be considered ‘trustworthy’, men stressed that it should come from authoritative and reputable sources, typically health professionals directly involved with their care (e.g. their primary care physician, hospital consultant or nurse). Some men acknowledged that their partner’s involvement would be integral to acting on dietary advice because “… many men are passive in the house … it needs to go through the women …” (P7).

### Healthcare professionals’ support for provision of dietary information to enhance survivorship

Both men and stakeholders described that men were rarely given dietary advice by their healthcare professional after their PC diagnosis. In the absence of convincing evidence in the literature of diet-PC links, some healthcare professionals indicated uncertainty about what information to provide patients, with most reporting that they did not routinely initiate discussion of dietary change with PC patients (see the ‘List of examples of health professionals’ opinions about the routine provision of dietary information to PC patients’). Most men, and their partners, reported that they would welcome and consider acting upon dietary advice should it become available, supporting findings from the in-depth interviews described above.

List of examples of health professionals' opinions about the routine provision of dietary information to PC patients

Uncertainty over what dietary information to provide

S1 (General Practitioner): … if someone came into me and said you know ‘well what’s the info doc?’ … I’d have to look that up because I don’t know and I wouldn’t know where to go for ready access to the data to support any discussions about that.

S6 (Consultant Urologist): … the short answer to it is yes I do give dietary advice to those that want it but probably I should be giving more advice to everybody but I haven’t done it up to recently you know because I’m not entirely sure what to say.

S6 (Consultant Urologist): I think there is some evidence and I’m sure it will come; it’s just a matter of time. Just because there isn’t any evidence doesn’t mean that you can’t, it doesn’t mean that it’s not right to offer dietary advice so I think the way you put it earlier was the best way is that here’s what we know, make your own mind up you know and I think that’s all that is is just empowering the patient, giving him the information and let them make the decision.

Benefits for general health

S1 (General practitioner): … modifying diet in any cancer process is beneficial and I would probably from my current stand point take that sort of tack in it talking about cancer in general rather than probably being informed enough to specifically talk about prostate cancer.

S3 (Dietician): … there isn’t any harm anything there so that would be quite good you know they’ve got other benefits as well. And it’s a really positive thing that people can do.

S6 (Consultant Urologist): When they do bring it up I say yea it’s a good idea to have a healthy diet because basically the sorts of the foods that we are talking about that are good for prostate cancer also are good for the other things that affects male health which is basically cardiac disease, so improving your diet anyway with, maybe, beneficial to prostate cancer although we haven’t actually certainly proven that yet is definitely going to be beneficial for general health, diabetes and heart disease and therefore I say to them go for it, it’s a good idea, it won’t do you any harm and it might just do some good.

S9 (Consultant Oncologist/Consultant Urological Surgeon): … it (dietary change) can’t be touted as ‘this will make your prostate cancer outcome better’. I think what it should be touted at until you get more information is that look, this appears to be useful, at the very worst it’s going to give you, you’re going to eat a healthier diet which is going be better for you anyway so and that’s a fairly pragmatic approach but I think that’s fairly sensible at the moment, and bearing in mind the whole selenium, vitamin E debacle, not a debacle but that’s actually a very good study that needed to be done.

Benefits for active surveillance patients

S6 (Consultant Urologist): We don’t know but it might be more relevant … to men for example who are on active surveillance for their prostate cancer who’ve got low risk low volume prostate cancer.

Role of the healthcare professional

S5 (Consultant Urological Surgeon): … the consultant who might be doing the operations … then people listen.

S5 (Consultant Urological Surgeon): … we’ve (consultants have) got a captive audience. GPs they see as generic filter don’t they?

S6 (Consultant Urologist): … if it comes from a doctor well they tend to listen a bit more sometimes.

Timing

S2 (PC survivor and PC support group organiser): they will have to go back, see their consultant to decide what sort of treatment they going to get and then I think it’s probably at that time that they should be told perhaps about diet ‘cause it’s all part and parcel of the recovery process hopefully.

S5 (Consultant Urological Surgeon): There’s some patients who you’d tailor it according to the situation; some are just so shocked by the diagnosis and their priority is to get through initial definitive treatment, get to grips with the diagnosis and what it means but they don’t consider that (diet) until much later.

Empowering patients

S6 (Consultant Urologist): … and I think that’s all that (dietary information) is, just empowering the patient, giving him the information and let them make the decision.

S9 (Consultant Oncologist/Consultant Urological Surgeon): … I think people should take more of a role in their own care.

A brief lay summary, optimally describing current evidence of foods/nutrients possibly causally related to PC or, at the very least, tips for general healthy eating were considered valuable by men, particularly if delivered in the context of a holistic ‘package of care’ and offered during the critical ‘teachable moment’ soon after diagnosis or around the time of treatment during which patients are more receptive to healthcare promotion activities. However, some participants did acknowledge that each patient should be considered individually, to avoid overloading them with information and causing increased anxiety around the time of diagnosis (see the ‘List of examples of health professionals' opinions about the routine provision of dietary information to PC patients’). Some healthcare providers also referred to the role dietary information may play in empowering patients and enabling them to be more involved in their own health (see the ‘List of examples of health professionals' opinions about the routine provision of dietary information to PC patients’). All parties agreed that the information would have greater impact if delivered face-to-face and preferably by a healthcare professional directly involved with the patient’s care, particularly the diagnostic/treatment process (such as an oncologist, urological consultant or surgeon). Inclusion of partners and/or family in the process of dietary information provision is desirable, due to their potential role as co-implementers in dietary change. Men requested that the source of the information (e.g. the WCRF recommendations) be acknowledged to offer reassurance that the information comes from a reputable and evidence-based source. Written information (e.g. an information leaflet) was preferred, enabling patients to refer back to it at their leisure and including links to sources of further information (e.g. websites). However, opportunity to discuss the information provided further and to obtain feedback on progress with dietary change was considered valuable.

## Discussion

This interview study explored views about diet including motivations for dietary change in men at elevated risk or diagnosed with PC. Findings indicated that, despite scepticism and uncertainty about diet-PC links, over half of men with PC made dietary changes that were generally ‘healthier’ or specifically ‘prostate healthy’ [3]. Dietary changes were perceived to bring psychological benefit, as a method of coping, enhancing survivorship or regaining control. PC treatment also influenced men’s perceptions of their disease state and whether they made dietary changes, with those undergoing non-radical treatment (AS/AM) more likely to perceive dietary changes a beneficial adjunct therapy than men who had surgery or radiotherapy. Men’s dietary information needs varied according to perceptions of treatment success but most men, including those at elevated risk of disease, expressed interest in receiving dietary advice. Men’s partners were considered integral to the success of dietary change. Overall, the provision of routine dietary advice to men diagnosed with PC was considered by all parties, including healthcare professionals, to be both feasible and appropriate in the context of a holistic ‘package of care’.

Men in this study were aged 52–75 and were invited for community-based PSA testing or diagnosed within routine clinical practice, and so are comparable to many contemporary PC patients. Qualitative methods enabled in-depth exploration of participants’ individual experiences and opinions. Interviews were conducted with 69 participants with a high response rate, including men with a range of diagnostic PSA levels, men at elevated risk or diagnosed with localised disease and undergoing different cancer treatments. Interviews with partners also allowed the influence of family on dietary decisions to be explored. Most participants were white and further exploration in other settings is desirable, particularly in men different cultural and socioeconomic groups to explore potential variations in health beliefs and dietary practices. Participants in the ProDiet intervention trial and the ProtecT treatment trial (who were asked to complete diet questionnaires pre- and post-diagnosis) may have had increased awareness of potential diet-PC links, though they were not routinely given dietary information within the study. The use of a qualitative method, involving direct (and mostly face-to-face) discussion between the participant and the researcher, may have increased the likelihood that participants would report dietary change, though no evidence was collected during this study that indicated that this actually occurred. Participants in research studies are more likely to practice health behaviours [5] and a control group was not included, although this study also included men diagnosed by their primary care physician and outside a trial context and their findings were comparable.

Many men were motivated to change their diet after PC diagnosis. This supports recent National Cancer Survivorship Initiative research highlighting that cancer patients want to know how to look after themselves, including how diet and lifestyle changes may help them to return to a ‘normal’ life [17,18] and previous surveys reporting that up to half of PC survivors change their diet to boost immunity and prevent recurrence [7,9,19] and that men on AS have a need for support services, including information about eating a healthy diet [20]. In particular, dietary changes reported by men in this study were often ‘heart healthy’ (e.g. increased fruit and vegetables, decreased fat and red meat). Recent findings from food questionnaire data from ProtecT showed that a PC diagnosis prompted one third of men to adopt healthier diets [21].

Research exploring motivations, obstacles and facilitators to dietary change among PC survivors outside of a trial context has previously been limited. This study supports findings from previous interview-based research indicating that men’s decision-making around changing their diet after a PC diagnosis is a complex process, where men construct a rationale for dietary change based on consideration of multiple factors, including their pre-cancer diet perceptions, diet-health understandings, perceptions of PC and the need to ‘do something’ [22]. The findings from this study further indicate that changes are often triggered after diagnosis but that the diagnosis itself may be only one contributing factor to initiating dietary change, serving as an ‘extra incentive’. This study also extends findings observed in survivors of other cancers, including prostate cancer, that interviewed men feel that dietary changes bring psychological benefit [6,23,24].

The importance of men’s partners in decision-making, optimising motivation and enhancing behaviour change was also emphasised, supporting evidence from men undergoing AS [20,25] and from other disease areas (e.g. cardiovascular disease, Type II diabetes) [10,11]. More recent research taking a ‘gender relational’ approach to explore health behaviour, has reinforced the stereotype that, in contrast to women, men are typically uninterested in ‘feminine’ health promotion practices, such as nutritional self-care, and instead adhere to socially-constructed masculine ideals about diet that may undermine rationales for dietary change [26,27]. However, the influence of men’s partners on dietary change is uncertain. This study demonstrated that men’s partners played an integral role in initiating and maintaining dietary change in male PC survivors, supporting the notion that the influence of a female partner may challenge masculine-derived rationales for dietary change (or lack thereof). We also found that, in contrast to the stereotype, some men were highly motivated to improve their diet and often assumed an active dietary leadership role. Dietary change may serve as a way of fostering a sense of confidence and control, both factors that are considered important to the stereotypical description of masculinity. Research into the potential influence of different treatments on men’s dietary decisions after a PC diagnosis has also been scarce. This study showed that type of cancer treatment may influence men’s dietary decisions post-diagnosis, with men on AM/AS reporting more interest in dietary factors for survivorship than men who received radical treatments. This supports findings from an interview study in which PC patients focused on diet as an adjunct therapy and self-management strategy to overcome AS-related anxiety, though there were no comparisons with radical treatments [25]. Recent research from the ProtecT trial further shows that men undergoing AM consumed more fruit/vegetable juice (including tomato juice) than those randomised to surgery [21].

Findings from this study can inform the design of dietary intervention trials for PC, providing further evidence that a cancer diagnosis offers a “teachable moment” during which health promotion activities may be most successfully initiated [19,26]. Previous cancer dietary intervention trials have been hampered by uncertainties over best methods for initiating and maintaining desired changes [28]. This study has identified a number of knowledge- and information-based barriers and facilitators to dietary change, which may be valuable in informing future trial design, most notably information provision and the role of men’s partners.

Improving understanding of dietary behaviour and information needs of men at elevated risk or diagnosed with PC may inform the provision of care for these patients but research exploring the views of key stakeholders, including men, their partners and healthcare professionals, has been lacking. The current absence of strong evidence for diet-PC links means that provision of disease-specific dietary advice can be neither systematic nor routine, although this study showed that men’s interest in dietary information was high and that patients may consider any dietary advice better than none. In particular, men undergoing non-radical treatment may benefit from focusing on dietary change to help them manage AM-related uncertainty [25,29]. More anxious patients may also want more information on a ‘prostate-friendly’ diet [30]. Thus there are opportunities for healthcare professionals to assist PC patients both clinically and psychologically by the routine integration of healthy eating advice into survivorship care plans [31,32], particularly for men embarking on AS/AM programmes who may have frequent monitoring appointments over a long period. This may be increasingly important with the increasing use of non-radical approaches in men with low-risk disease worldwide [20] and the lack of other actions that can be taken [33,34]. Targeting dietary information at both men and their partners may encourage shared responsibility and strengthen men’s rationales to make positive dietary changes.

## Conclusions

The diagnosis of prostate cancer (PC) can prompt men to adopt a healthier diet, which may improve quality of life and clinical outcomes. However, men’s views about dietary change in PC survivorship are largely unknown. Many men make positive dietary changes after PC diagnosis, which are perceived to bring psychological and general health benefits and could help future dietary intervention trials. Men’s partners have an important role in supporting them to make positive dietary changes, but uncertainty around the potential benefits of healthy eating after diagnosis may prevent dietary change. In particular, men and their partners desire more and better dietary information that may support PC survivorship, particularly among those embarking on active surveillance/monitoring programmes. There are opportunities for healthcare professionals to support PC patients both clinically and psychologically by the routine integration of healthy eating advice into survivorship care plans.

## Abbreviations

AM: Active monitoring; AS: Active surveillance; AS study: Active Surveillance Study; FFQ: Food Frequency Questionnaire; PC: Prostate cancer; PRODIET: Prostate and Diet Study (International Standard Randomised Controlled Trial Number ISRCTN95931417); PROTECT: Prostate testing for cancer and Treatment study (International Standard Randomised Controlled Trial Number 20141297); PSA: Prostate specific antigen; REC: Research Ethics Committee.

## Competing interests

The authors declare that they have no competing interests.

## Authors’ contributions

KA participated in the conception and design of the study, coordinated the study, collected the data, conducted the analyses and drafted the manuscript. JD participated in the conception and design of the study and helped to draft the manuscript. JH contributed to the data analyses and commented on the final manuscript. DN and FH participated in the conception and design of the study and commented on the final manuscript. CP participated in the design of the study, assisted with the coordination of the study and commented on the final manuscript. JW contributed to the data analyses and commented on the final manuscript. AL participated in the conception and design of the study, assisted with the coordination of the study and helped to draft the manuscript. All authors read and approved the final manuscript.

## Pre-publication history

The pre-publication history for this paper can be accessed here:

http://www.biomedcentral.com/1471-2296/15/81/prepub
